# A computerized diagnostic model for automatically evaluating placenta accrete spectrum disorders based on the combined MR radiomics-clinical signatures

**DOI:** 10.1038/s41598-022-14454-w

**Published:** 2022-06-16

**Authors:** Hao Zhu, Xuan Yin, Haijie Wang, Yida Wang, Xuefen Liu, Chenglong Wang, Xiaotian Li, Yuanyuan Lu, Guang Yang, He Zhang

**Affiliations:** 1 Department of Obstetrics, Obstetrics and Gynecology Hospital, Fudan University, Shanghai, People’s Republic of China; 2 Department of Radiology, Obstetrics and Gynecology Hospital, Fudan University, Shanghai, People’s Republic of China; 3 Shanghai Key Laboratory of Magnetic Resonance, East China Normal University, Shanghai, People’s Republic of China; 4 Department of Radiology, Shanghai First Maternity and Infant Health Hospital, School of Medicine, Tongji University, Shanghai, People’s Republic of China

**Keywords:** Medical research, Outcomes research

## Abstract

We aimed to establish a computerized diagnostic model to predict placenta accrete spectrum (PAS) disorders based on T2-weighted MR imaging. We recruited pregnant women with clinically suspected PAS disorders between January 2015 and December 2018 in our institution. All preoperative T2-weighted imaging (T2WI) MR images were manually outlined on the picture archive communication system terminal server. A nnU-Net network for automatic segmentation and the corresponding radiomics features extracted from the segmented region were applied to build a radiomics-clinical model for PAS disorders identification. Taking the surgical or pathological findings as the reference standard, we compared this computerized model’s diagnostic performance in detecting PAS disorders. In the training cohort, our model combining both radiomics and clinical characteristics yielded an accuracy of 0.771, a sensitivity of 0.854, and a specificity of 0.750 in identifying PAS disorders. In the testing cohort, this model achieved a segmentation mean Dice coefficient of 0.890 and yielded an accuracy of 0.825, a sensitivity of 0.830 and a specificity of 0.822. In the external validation cohort, this computer-aided diagnostic model yielded an accuracy of 0.690, a sensitivity of 0.929 and a specificity of 0.467 in identifying placenta increta. In the present study, a machine learning model based on preoperative T2WI-based imaging had high accuracy in identifying PAS disorders in respect of surgical and histological findings.

## Introduction

Placenta accrete spectrum (PAS) is a general term used to describe abnormal trophoblastic invasion into the myometrium of the uterus. The incidences of various risk factors, such as cesarean sections and abortions, are gradually increasing worldwide. The overall prevalence was 1/695-731 over the past ten years^1,2^. At present, there are no large-scale epidemiological data on PAS occurrence in China, although a high cesarean section rate has been reported^3^. At present, the pathogenesis of PAS and the specific pathophysiological process are still unclear. The occurrence of PAS may be attributed to the combined effects of one or more pathological factors, such as the absence of basal decidua, local oxygen tonal abnormality, abnormal vascular remodeling, and the excessive invasion of trophoblast cells^4^. PAS can lead to a variety of complications, such as hemorrhage during or after delivery, disseminated intravascular coagulation, renal failure, and venous thrombosis, which may lead to maternal or fetal death in severe cases^5^. Early diagnosis is the upmost effective method to prevent postpartum hemorrhage and reduce maternal mortality.

PAS disorders can be diagnosed by clinical symptoms and signs, laboratory examination, ultrasound (US) or magnetic resonance imaging (MRI)^6–8^. US examinations are cheap, easy to perform and widely applied in the clinical setting for PAS diagnosis. MR examination has the technical advantages of high soft tissue resolution and multidirectional and multisequence imaging and is not affected by the position of intestinal gas, bones or the placenta, which can provide more information for the diagnosis of PAS^9^. MRI is an important complementary tool for US in both the diagnosis and staging of PAS disorders. The sensitivity and specificity of MRI diagnosis of PAS range from 87 to 100% and 97 to 99%, respectively^6,10,11^, and the accuracy of its diagnosis before invasive surgical procedures is highly dependent on radiologists’ expertise^10,12^. Minimizing the knowledge differences across various institutions could help improve the accuracy of the preoperative diagnosis of PAS with imaging modalities in primary hospitals, supply timely and reasonable referrals and reduce the potential maternal mortality rate. In recent years, machine learning based on imaging data has provided convincing results for various tasks in medicine. It was reported that MR-based radiomics could help improve the preoperative estimation of postpartum hemorrhage volume related to PAS disorders with an accuracy of 68.1% in the validation group^13^. However, similar studies are still alarmingly limited^14^.

The purpose of this study was twofold: (1) to train a nnU-Net network to automatically sketch out placental tissue on sagittal T2-weighted imaging and (2) to establish a radiomics-clinical combined model for the prediction of PAS disorders and then to calculate its diagnostic performance, taking surgical and/or pathological findings as the reference standard.

## Materials and methods

### Patients

**Figure 1:**
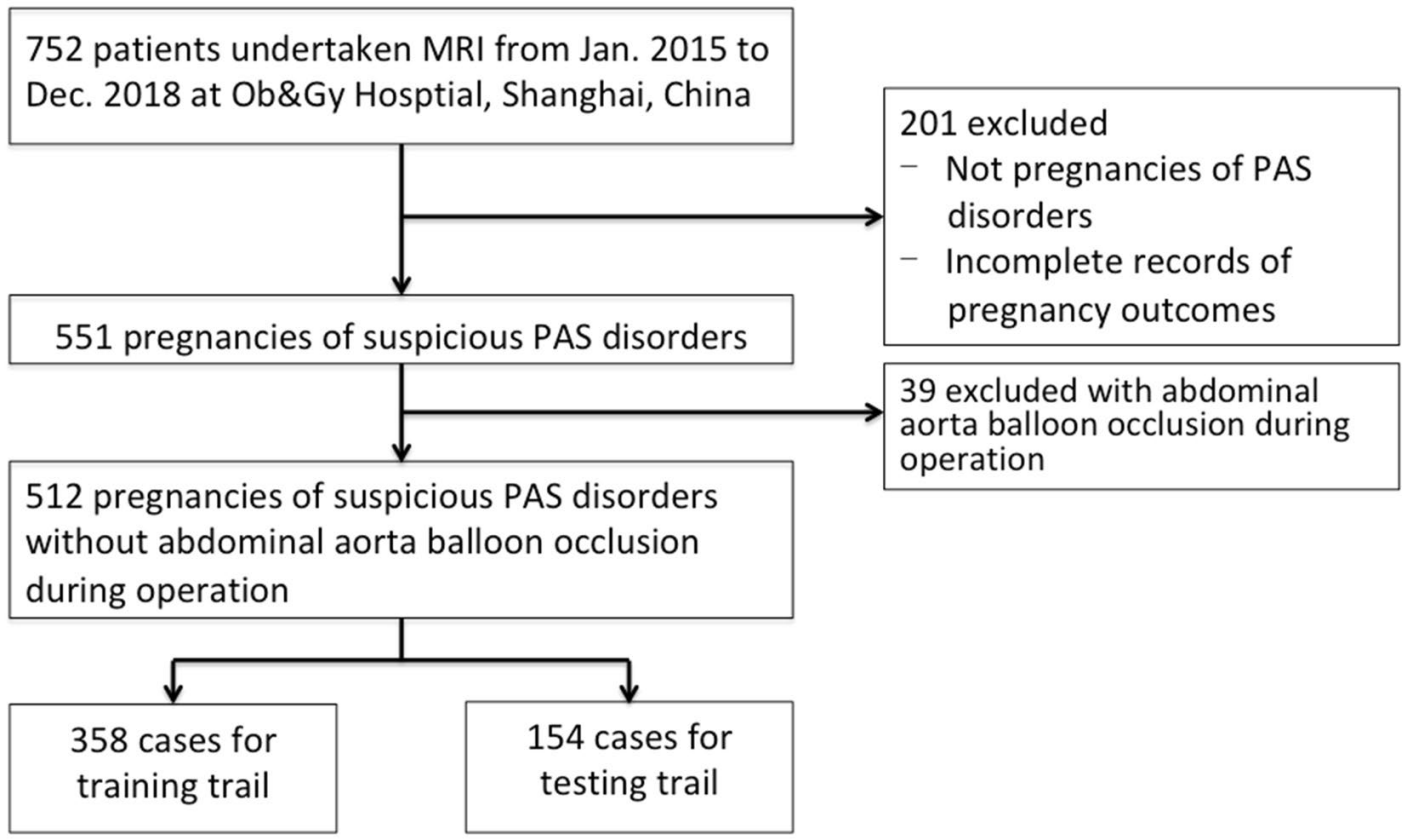
The flowchart of the recruited samples in this study.

Our institutional review board approved this retrospective study. All methods were performed in accordance with the relevant guidelines and regulations. From January 2015 to December 2018, data from 752 patients who underwent MRI, including 551 pregnant women with clinically suspected PAS disorders (either by previous US or external MR reports), were retrospectively retrieved from the PACS at our institution. We excluded 39 of them who had undergone abdominal aorta balloon occlusion surgery before delivery. Finally, 512 pregnant women were included (Fig. 1). We divided the included pregnancies into the following five categories (scores from 0 to 5 points) based on placental position: 0, normal placental position; (1) low-lying placenta; (2) marginal placenta previa; (3) partial placenta previa; (4) central placenta previa; and (5) pernicious placenta previa.

### MR examination, imaging reading and lesion segmentation

In our hospital, MR examination was performed using a 1.5-Tesla MR unit (Magnetom Avanto, Siemens). In the external hospital, one MR scan unit was a 3.0-Tesla machine (Ingenia 3.0 T, Philips), and another was a 1.5-Tesla MR unit (Optima MR 360, GE). The detailed scanning parameters for each unit are summarized in Supplementary Table 1. The diagnosis for placental implantation on MRI was established based on the previous well-described criteria^8,15^. First, two observers (each had more than 7 years of PAS diagnosis on MRI) blinded to the US and surgical results analyzed all the MRI datasets of each participant independently on the PACS terminal server. Confidence in identifying the status of placenta accreta spectrum was assessed using a five-point scale as follows: ‘5’, definitely present; ‘4’, probably present; ‘3’, uncertain; ‘2’, probably absent; and ‘1’, definitely absent. Second, all conclusions required a consensus agreement between the two observers. All visible placental tissue was examined by an experienced radiologist (H.Z.) on sagittal T2WI using ITk-SNAP software (http://www.itksnap.org/pmwiki/pmwiki.php?n=Main.HomePage).

### Semantic segmentation and radiomic feature extraction

**Figure 2:**
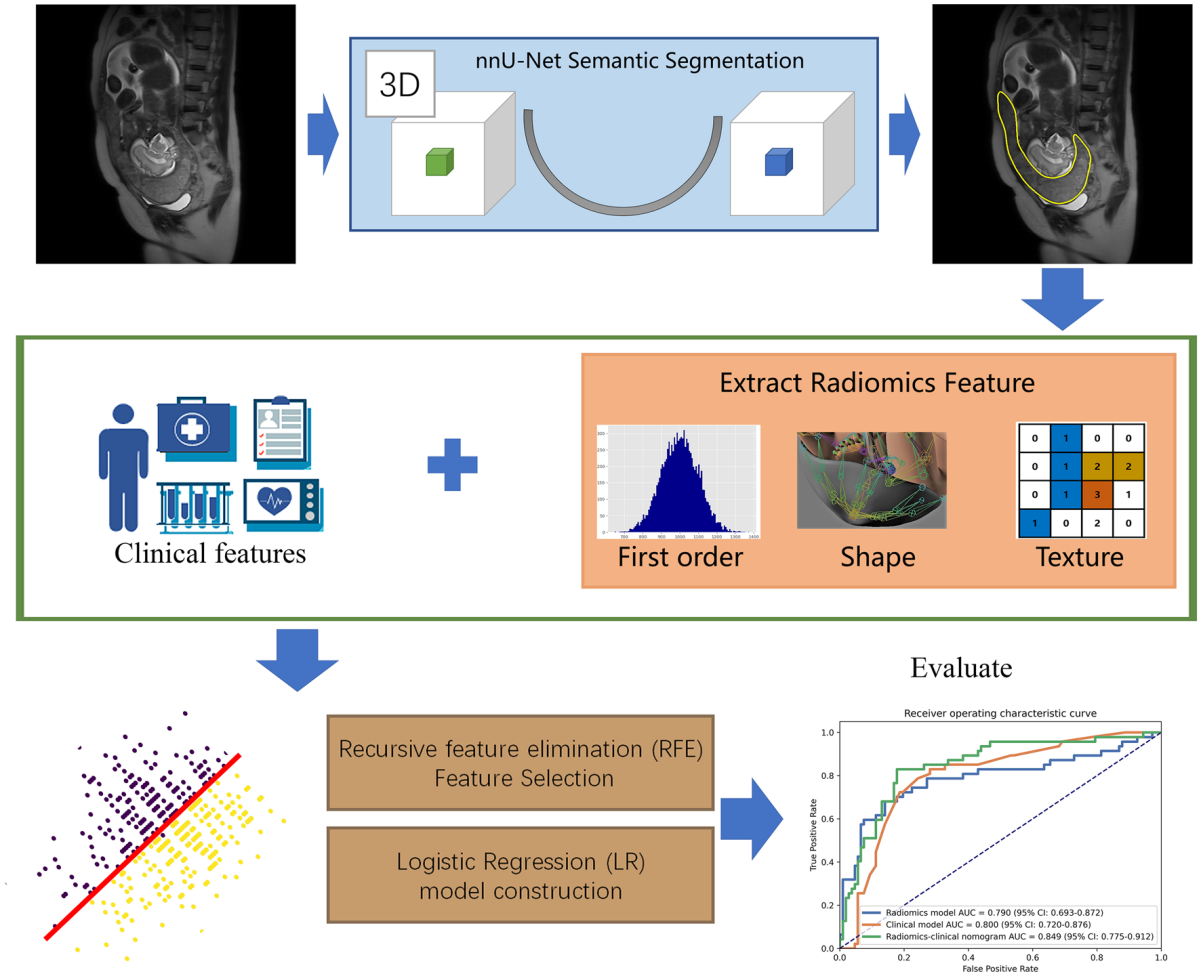
A trained nnU-Net model was utilized to automatically segment placenta region on sagittal T2WI. The radiomics features were extracted from the segmented region and combined with clinical features to build a machine learning model for prediction of PAS disorders.

The pipeline of the imaging process is shown in Fig. 2. A semantic segmentation three dimensional (3D) nnU-Net model was trained by the radiologist, who labeled the placental region to segment the placenta used for radiomics feature extraction^16^. nnU-Net can automatically adapt to arbitrary datasets and take full advantage of the characteristics of the dataset to outperform many U-Net-based models and has been used successfully in 3D biomedical image segmentation. We used a combination of cross entropy loss and dice loss as the loss function. The stochastic gradient descent algorithm was used as the optimizer with an initial learning rate of 0.01, a momentum of 0.9 and a weight decay of 0.005. In brief, a total of 107 features were extracted from each placental region, including the following: (1) 14 shape features; (2) 18 first-order statistical features; and (3) 75 texture features, including the gray level cooccurrence matrix (GLCM), gray-level dependence matrix (GLDM), gray-level run-length matrix (GLRLM), gray-level size zone matrix (GLSZM) and neighborhood gray-tone difference matrix (NGTDM). Feature extraction and model building were implemented with the open-source software FeatureExplore^17^.

### Dataset split

We stratified the dataset based on the archiving time into a training cohort (110 women with PAS disorders versus 248 with normal placentas, from 2015 to 2017) and a testing cohort (47 women with PAS disorders versus 107 with normal placentas, 2018). In the external hospital, 11 pregnancies with a 3.0 T scanner and 17 pregnancies with a 1.5 T MR scanner were treated (15 women with PAS disorders/13 with normal placentas, 2020) as an external validation cohort.

### Radiomics-clinical nomogram construction

The radiomics score (rad-score) was calculated for each patient cohort by the linear combination of the selected features in the best radiomics signature. A clinical Logistic Regression (LR) model based on clinical characteristics, such as placental position, maternal age, and gravida, was also built. Furthermore, multivariable logistic regression analysis was developed with the rad-score and clinical variables. A radiomics-clinical nomogram was constructed based on multivariable logistic analysis in the training cohort to quantitatively predict the risk of PAS disorders.

### Performance comparison of both the models and radiologists for PAS diagnosis

Taking the surgical or histopathological findings as the gold standard, the accuracy (ACC), sensitivity (SEN), specificity (SPE), positive predictive value (PPV) and negative predictive value (NPV) of both the computer model and radiologists for PAS diagnosis were separately calculated and compared. Decision curve analyses (DCAs) were used to determine the effectiveness of the combined radiomics-clinical computerized model. A t test or Mann–Whitney test was performed to assess the differences between the two cohorts. A *P* value < 0.05 was regarded as statistically significant. Receiver operating characteristic (ROC) curves and areas under the curve (AUCs) were used to evaluate the diagnostic performance of the various methods. R software (version 4.0.4, http://www.R-project.org) was used to perform statistical analyses and plot the nomogram.

### Ethics approval

Our institutional review board approved the study, and the requirement for the informed consent of all participants was waived. In this article, institutional review board was review board of Obstetrics and Gynecological hospital, Medical College, Fudan University.

### Consent to participate

The requirement for the informed consent of all participants was waived (IRB No.2020-138). The signed consent for further MR scan was obtained from each pregnancy when have indeterminate anomalies on screening ultrasound. This consent will comprehensively tell each pregnancy that indications, contraindications, advantages, limitations and potential medical use on MR imaging.

### Consent to publication

**Figure 3:**
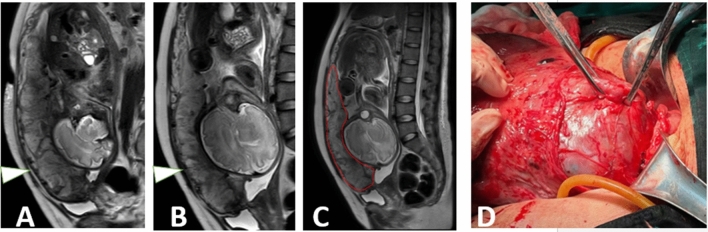
A 38-year-old gravid woman with pernicious placenta previa at 35.1 GW. The normal interface (arrowhead) between normal (**A**) and increta (**B**) placental interface were displayed on sagittal T2WI. The computerized model automatically drew the contour of placenta and gave the prediction probability of 0.937 in placenta increta (**C**). The surgical findings showed the perforating vessels along the uterine serosa (**D**).

**Figure 4:**
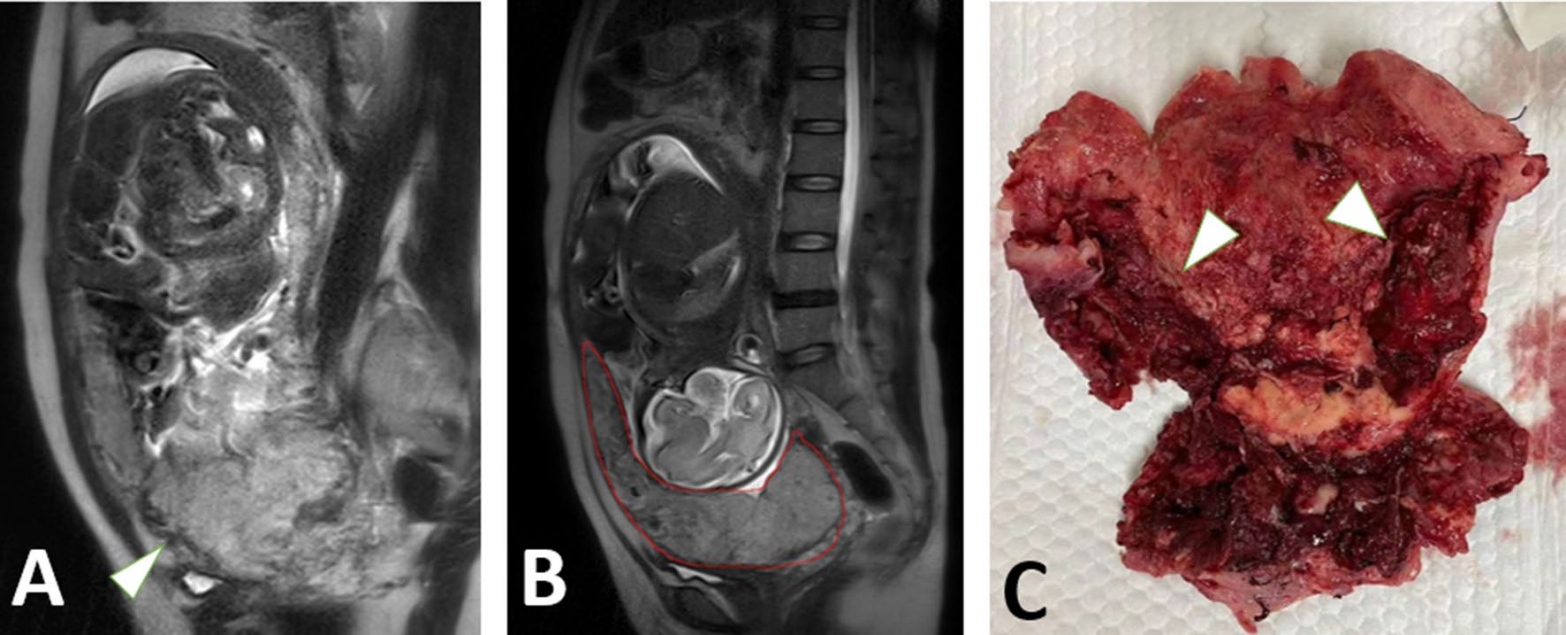
A 35-year-old gravid woman with placenta previa at 34.3 GW. The placenta increta (arrowhead) were clearly displayed at the lower segment of the uterus on sagittal T2WI (**A**). The computerized model automatically drew the contour of placenta and gave the prediction probability of o.98 in placenta increta (**B**). The surgical specimen (arrowhead) disclosed the invasive placentation near the lower uterine segment (**C**).

**Figure 5:**
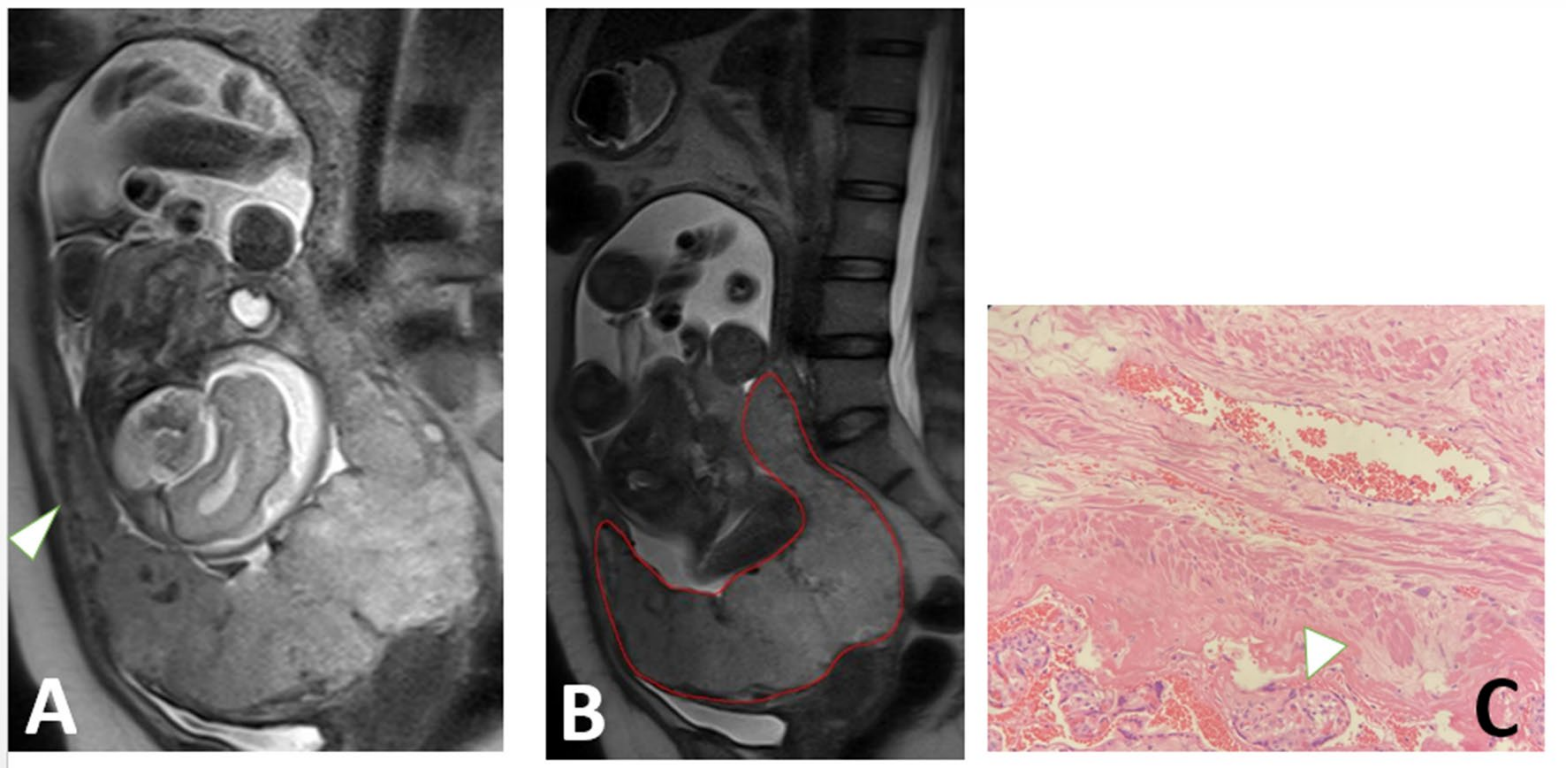
A 35-year-old gravid woman with placenta previa at 34.2 GW. The placenta increta (arrowhead) were clearly displayed at the lower uterine segment on sagittal T2WI (**A**). The computerized model automatically drew the contour of placenta and gave the prediction probability of 0.877 in placenta increta (**B**). The histological picture disclosed the placenta villi (arrowhead) among the muscular vessels in the uterus (HE × 100, **C**).

Verbal informed consent for publication was obtained from the pregnancies to usage these clinical and imaging data (Figs. 3, 4, 5 and 7).

## Results

### Baseline characteristics

In the present study, the patients were 21 to 48 years of age and between 18 and 41 weeks of gestation. The average gravida and parity were 3.63 and 1.83, respectively. Among them, 397 (77.54%) had no history of cesarean section, and 115 (22.46%) had undergone a cesarean section 1 to 4 times (Table 1). Ninety-four (18.36%) women were diagnosed with diabetes mellitus (including pregestational diabetes mellitus and gestational diabetes mellitus), 31 (6.05%) were diagnosed with hypertensive disorders (including gestational hypertension, preeclampsia, and chronic hypertension complicating pregnancy), 19 (3.71%) had undergone in-vitro fertilization embryo transplantation, 20 (3.91%) were diagnosed with hypothyroidism, and 8 (1.56%) were twin pregnant women. A total of 102 (19.93%) women underwent vaginal delivery, including 3 (0.59%) who underwent operative vaginal delivery; 409 (79.88%) underwent cesarean section; and 1 (0.20%) underwent spontaneous abortion. In our data, the mean estimated blood loss (EBL) volume during surgery was 308.56 ± 155.26 ml in pregnancies with a normal placenta, 533.00 ± 1053.29 in those with marginal placenta previa, 675.00 ± 599.40 in those with partial placenta previa, 823.39 ± 1041.95 in those with central placenta previa, 1926.10 ± 1783.91 in those with pernicious placenta previa, and 503.04 ± 481.69 in those with low lying placenta.

**Table 1:** Baseline characteristics of the included 512 pregnancies of clinically suspected PAS disorders.

Age, years (mean ± SD)	33.22 ± 4.61 (21–48)
Gravida (mean ± SD)	3.63 ± 2.05 (1–13)
Para (mean ± SD)	1.83 ± 0.75 (1–6)
GW, week (mean ± SD)	37.62 ± 2.36 (18.3–41.4)
**History of cesarean section, n (%)**	
0	397 (77.54)
1	73 (14.26)
2	40 (7.81)
3	1 (0.20)
4	1 (0.20)
**Maternal comorbidities, n (%)**	
Diabetes mellitus	94 (18.36)
Hypertensive disorders	31(6.05)
IVF-ET	19 (3.71)
Hypothyroidism	20 (3.91)
Twin pregnancy	8 (1.56)
**Delivery, n (%)**	
Vaginal	99 (19.34)
Operative vaginal	3 (0.59)
Cesarean section	409 (79.88)
Others (abortion)	1 (0.20)
**EBL, ml (mean ± SD)**	
*Placenta position, n: 0 (268)	308.56 ± 155.26 (130–1200)
1 (30)	533.00 ± 1053.29 (200–6000)
2 (8)	675.00 ± 599.40 (200–2000)
3 (115)	823.39 ± 1041.95 (200–7280)
4 (68)	1926.10 ± 1783.91 (200–10,000)
5 (23)	503.04 ± 481.69 (200–2500)

EBL, estimated blood loss; IVF-ET, in-vitro fertilization embryo transplantation.

### Performance of semantic segmentation and radiomics signatures

The trained 3D nnU-Net model obtained a mean dice coefficient of 0.890 in the testing cohort. The segmentation was in good agreement with the radiologists' labels (Figs. 3, 4 and 5). From the subgroups, 8 shape features, 1 first-order feature and 9 texture features were selected because of the higher cross-validation AUC. Among the T2WI radiomics signatures, 6 radiomics features were retained in the final LR model with an AUC of 0.792 (95% confidence interval (CI): 0.736–0.844) in the training cohort and 0.790 (95% CI: 0.698–0.876) in the testing cohort.

### Clinical data analysis

There were significant differences in some clinical variables between those with PAS disorders and normal placentas (Table 2). The clinical-radiological signature used age, placental location, and gravida selected by the recursive feature elimination (RFE) algorithm and achieved an AUC of 0.807 (95% CI: 0.759–0.857) in the training cohort and 0.800 (95% CI: 0.743–0.850) in the testing cohort.

**Table 2:** Summaries of clinical characteristics in the training, testing and external testing cohort.

Characteristics	Training			Validating			Testing		
	Normal (n = 248)	PAS disorders (n = 110)	*P* value	Normal (n = 107)	PAS disorders (n = 47)	*P* value	Normal (n = 13)	PAS disorders (n = 15)	*P* value
Age, years (mean ± SD)	33.0 ± 4.6	33.7 ± 4.3	0.180	33.0 ± 4.5	33.7 ± 5.0	0.378	32.7 ± 3.9	36.4 ± 3.3	0.015
Gravida (mean ± SD)	3.5 ± 2.1	4.1 ± 1.9	0.100	3.6 ± 2.0	3.5 ± 1.8	0.145	2.1 ± 1.2	2.7 ± 2.0	0.656
Para (mean ± SD)	1.8 ± 0.8	1.8 ± 0.6	0.407	1.8 ± 0.8	1.7 ± 0.7	0.570	0.7 ± 0.6	0.8 ± 0.8	0.713
Placental position			< 0.001			< 0.001			0.004
Normal	164	21		76	7		15	2	
Low lying placenta	19	4		5	2		0	0	
Marginal placenta previa	3	3		1	1		0	1	
Partial placenta previa	38	41		15	21		0	0	
Complete placenta previa	12	37		4	15		2	4	
Pernicious placenta previa	12	4		6	1		0	6	

*P* value of all characteristics are calculated by one of independent-samples t-test, Mann–Whitney U-test or chi-squared test based on their distribution.

### Performance of the radiomics-clinical nomogram and comparison with radiologists’ performance

**Figure 6:**
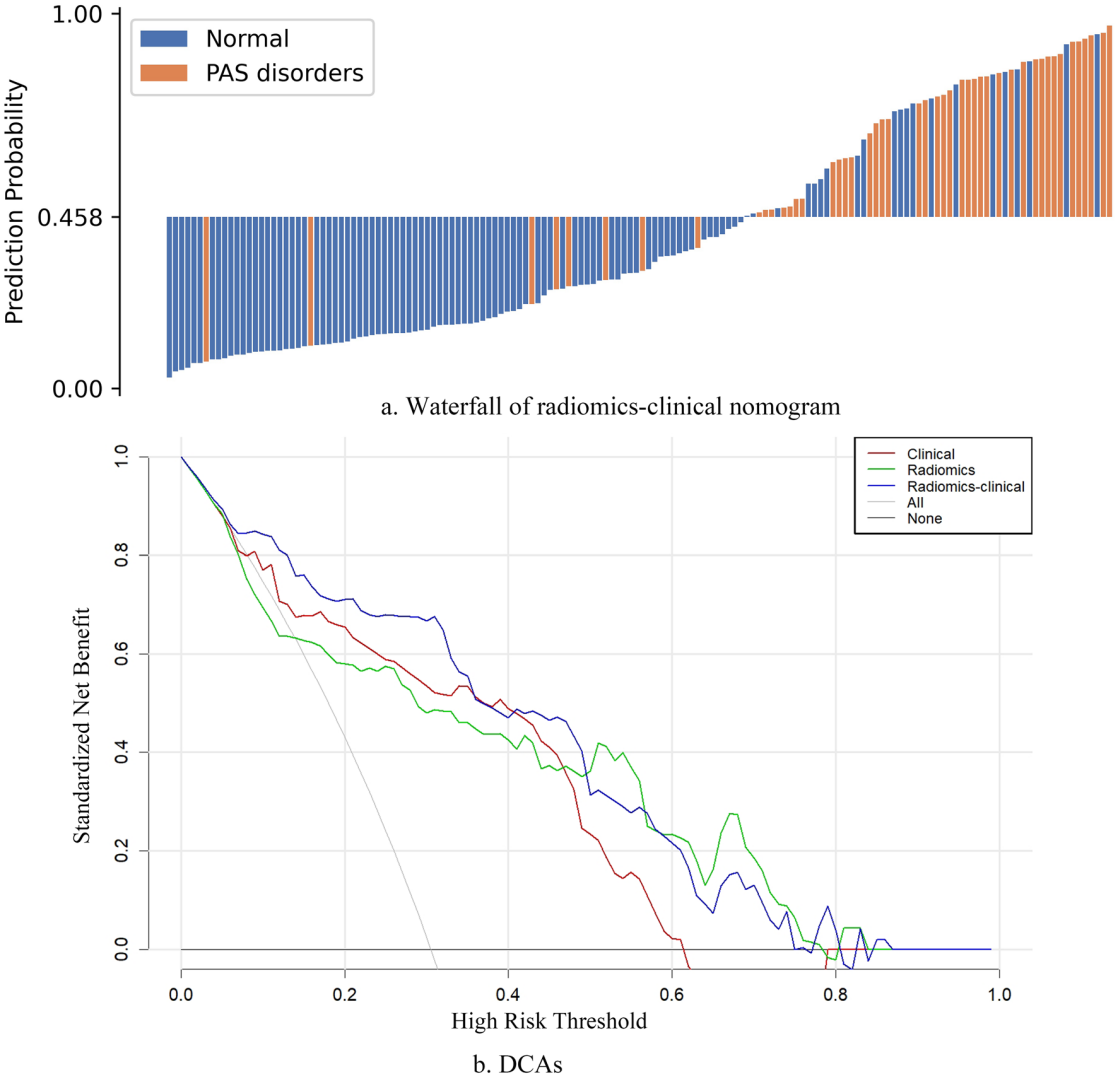
(**a**) The waterfall plot for the distribution of prediction probability of radiomics-clinical nomogram in the testing cohort. The cutoff value of 0.488 was defined based on the Youden index in the training cohort. (**b**) DCAs for radiomics signature (red line), clinical-radiological signature (green line) and radiomics-clinical nomogram (blue line). The “All” line is made with the assumption that all patients are poor prognosis.

**Figure 7:**
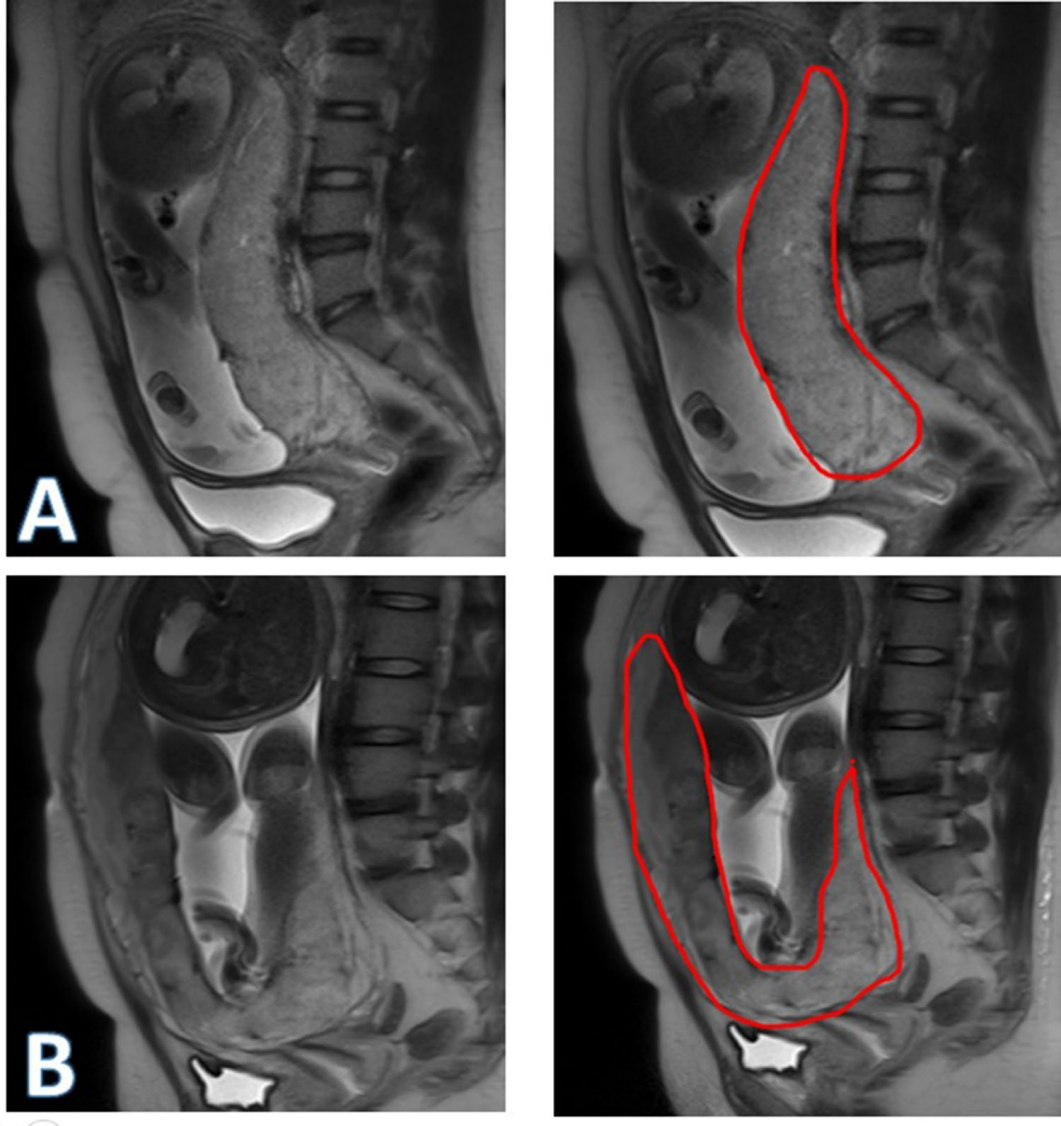
A 29-year-old gravid woman with placenta previa at 34.5 GW (**A**). Both the raw image and the segmented area were displayed on sagittal T2WI. In this case, the model gave the prediction probability of 0.264 in PAS status, representing a false-negative error. A 28-year-old gravid woman with complete placenta previa at 37.2 GW (**B**). The raw image and the corresponding segmented image were also displayed. In this case, the model gave the prediction probability of 0.744 in PAS status, representing a false-positive error.

The diagnostic performance from the radiomics features extracted from manual segmentation by radiologists was similar to those extracted from the nnU-Net's segmentation (AUC: 0.847 vs 0.849) for PAS identification. The radiomics-clinical nomogram was constructed based on LR using the rad-score and clinical characteristics (Supplementary Fig. 1). The combined model achieved the best performance with AUCs of 0.833 (95% CI: 0.780–0.882) and 0.849 (95% CI: 0.778–0.914) in both the training and testing cohorts, respectively. The statistical analysis for the clinical/radiomics/radiomics-clinical nomogram in the two cohorts is shown in Table 3. The waterfall plot of the testing cohort with an optimal cutoff value of 0.458 for the distribution of the prediction probability is shown in Fig. 6. The DCAs curve indicated that the net benefit of the nomogram was better than the other models when the threshold was in the range between 0.1 and 0.5. The radiomics signature had a higher positive prediction rate when the threshold was greater than 0.5. In external validation, the nomogram performed similarly to the testing cohort with an AUC of 0.862 (95% CI: 0.697–0.980). In the testing group, this trained model showed better discriminative ability than the radiologists (AUC: 0.849 versus 0.744, Table 4). For our studied cases, either small invasion signs on MRI or large interface area between placenta and myometrium (for example, both anterior and posterior uterine wall involved) mostly attributed to the model’s error identification (Fig. 7).

**Table 3:** The diagnostic performance of clinical, radiomics and nomogram models in detecting PAS disorders in training, validation and testing group.

Cohort	AUC (95% CI)	ACC	SEN	SPE	PPV	NPV
**Training (N = 358)**						
Clinical	0.807 (0.759–0.857)	0.776	0.736	0.794	0.614	0.872
Radiomics	0.792 (0.736–0.844)	0.729	0.800	0.698	0.540	0.887
Radiomics-clinical	0.833 (0.780–0.882)	0.771	0.854	0.750	0.605	0.919
**Validation (N = 154)**						
Clinical	0.800 (0.743–0.850)	0.768	0.727	0.786	0.602	0.867
Radiomics	0.790 (0.698–0.876)	0.805	0.681	0.860	0.681	0.860
Radiomics-clinical	0.849 (0.778–0.914)	0.825	0.830	0.822	0.672	0.917
**Testing (N = 28)**						
Radiomics-clinical	0.862 (0.697–0.980)	0.690	0.929	0.467	0.619	0.875

**Table 4:** The diagnostic performance comparison between radiologists and computers in prediction placenta accreta or increta in testing group (N = 154).

	AUC	ACC	SEN	SPE	PPV	NPV
Radiologists	0.744	0.753	0.532	0.850	0.610	0.805
MR-based Radiomics	0.790	0.805	0681	0.860	0.681	0.860
MR-based Nomogram	0.849	0.825	0.830	0.822	0.672	0.917

## Discussion

With the comprehensive opening of the "third child" policy in China, the number of people who get married at a later age and have children at a later age increases. It can be speculated that the incidence of placental implantation owing to cesarean surgery in China may further increase. In our study, we designed a computerized diagnostic model for evaluating PAS from a large cohort sample from a single institution. Our model yielded an AUC of 0.849 and an ACC of 0.825 in detecting invasive placentation in the testing group. In the external validation group, our model also provided the competitive diagnostic performance in detecting PAS. To our knowledge, this is the first reported study focusing on PAS disorder identification with machine learning techniques using T2WI images.

Several meta-analysis studies concluded that MRI yielded a SEN of 94.4%, 100% and 86.5% and a SPE of 98.8%, 97.3%, and 96.8% for the detection of placenta accreta, increta and percreta, respectively^6,7^. However, in recently published articles, these values varied with an SEN from 66 to 100% and an SPE from 71% to 76.9%^10,12^. Our study’s results also did not reach the high diagnostic performance level based on MRI knowledge. Some reasons are explained as follows. First, radiologists’ experiences play a vital role in the interpretation of PAS. In a recent study, the authors disclosed that abdominopelvic MRI experience without specific placental MRI experience did not improve diagnostic performance in PAS diagnosis^10^. Knowledge of PAS-related MRI findings definitely varies among various institutions, even in tertiary centers. Second, in our study, the final results included both surgical and histological findings. We could imagine that some objective judgments of PAS diagnosis from individual obstetricians may be incorrect. In our study, we did not use postpartum hemorrhage volume^13^ as the reference standard because, on the one hand, the blood loss volume in pregnant women, who underwent previous abdominal aorta balloon occlusion in case massive bleeding occur, may be inaccurate; on the other hand, pregnancies with either accreta or increta placenta diagnosed by obstetricians may not have massive blood loss but may still require some necessary treatments or follow-up after delivery. We believe that our present gold standard is similar to real scenarios where placenta accreta or increta is the greatest concern for obstetricians before invasive procedures.

Herein, we designed a pipeline algorithm aimed at confirming PAS disorders. Our study had several characteristics that were different from those of previous studies^13,14,18^. A trained 3D nnU-Net model was utilized to automatically segment the placental region on T2WI images. Compared with previous radiomics studies^13^, automatic segmentation of targeted regions could reduce the subjectivity, workload of radiologists and negative impact of interreader variance on predictions. This variance is relatively slight due to the good tissue contrast between the placenta and the myometrium on T2WI images. Our present results showed that the radiomics-clinical model achieved a satisfying performance in PAS disorder identification. The radiomics texture features reflect intraplacental heterogeneity, which may indicate physiological changes, such as fibrin deposition. Clinical features, including placenta location, maternal age, and gravida and parity, played an equally important role. Compared to one study using deep learning (DL) method to predict PAS disorders^14^, our study did not apply DL method to extract high-level imaging features. The trained DL network here was only utilized to segment the placental region on MRI. In our opinion, the cascaded modeling process consists of many model building steps including model training, DL feature extraction and combine both the deep learning and radiomics-extracted signatures to get the predicted point and predict placenta invasion. Many combinations of algorithms and hyper-parameters need to be explored to get the best results, which make the process vulnerable to information leaking and overfitting, especially when the studied sample size is small. We compared the model’s prediction capability with the radiologists’ performance at the two-task level. The model’s performance outperformed or was equal to the radiologists’ performance in PAS status evaluation. The trained model can process the unsegmented data and get the result within two minutes for each case, which help radiologists, especially less experienced residents, make a correct conclusion with more confidence. In our institution, this computerized model will help our physicians to establish the risk scores for each suspicious PAS pregnancies at the multidisciplinary treatment discussion. Further, this standardized-diagnostic model may also improve the diagnostic confidence and accuracy on PAS identification on MRI through telemedicine, especially for less-trained radiologists in some primary institutions where equip with MR unit. Although, artificial intelligence (represented by machine learning) technology can help physicians to tackle problems effectively, precisely at present; such technology should better incorporate with modern medicine in order to overcome its inherent shortcomings and limitation in the future^19^.

Our study has several limitations. First, we train this computerized model using MRI in our single institution, and the external validation samples are limited. The true diagnostic performance still needs to be validated in a larger cohort. Second, in the present study, we only used a single sequence of MRI (sagittal T2WI) to reconstruct the assisted diagnostic model. Theoretically, more selected sequences could help improve the estimated performance to some extent. Third, in this study, 1.5 T MR equipment was applied. Although it has not yet been well evaluated for intrauterine examinations, 3.0 T in utero MR imaging with a high signal-to-noise ratio and fast scanning protocols may improve image resolution^20^.

## Conclusion

In the present study, a machine learning model based on preoperative T2WI-based imaging had high accuracy in identifying PAS disorders in respect of surgical and histological findings.

## Supplementary Information

Supplementary Information.

## Data Availability

The authors declare that all data supporting the findings of this study are available within the article.

## Abbreviations

DWI: Diffusion-weighted magnetic resonance imaging
EBL: Estimate blood loss
MRI: Magnetic resonance imaging
US: Ultrasound
GW: Gestational week
PAS: Placenta accrete spectrum
PACS: Picture archiving and communication system
DCAs: Decision curve analyses
ROC: Receiver operator curve
AUC: Area under the curve
3D: Three dimensional
LR: Logistic regression
RFE: Recursive feature elimination
DL: Deep learning
