# Author Correction: A computerized diagnostic model for automatically evaluating placenta accrete spectrum disorders based on the combined MR radiomics-clinical signatures

**DOI:** 10.1038/s41598-022-16140-3

**Published:** 2022-07-11

**Authors:** Hao Zhu, Xuan Yin, Haijie Wang, Yida Wang, Xuefen Liu, Chenglong Wang, Xiaotian Li, Yuanyuan Lu, Guang Yang, He Zhang

**Affiliations:** 1 Department of Obstetrics, Obstetrics and Gynecology Hospital, Fudan University, Shanghai, People’s Republic of China; 2 Department of Radiology, Obstetrics and Gynecology Hospital, Fudan University, Shanghai, People’s Republic of China; 3 Shanghai Key Laboratory of Magnetic Resonance, East China Normal University, Shanghai, People’s Republic of China; 4 Department of Radiology, Shanghai First Maternity and Infant Health Hospital, School of Medicine, Tongji University, Shanghai, People’s Republic of China

Correction to: *Scientific Reports* https://doi.org/10.1038/s41598-022-14454-w, published online 16 June 2022

The original version of this Article contained an error, where an erroneous figure was uploaded instead of a flowchart of the recruited samples in this study.

The original Figure 1 and accompanying legend appear below.

**Figure 1:**
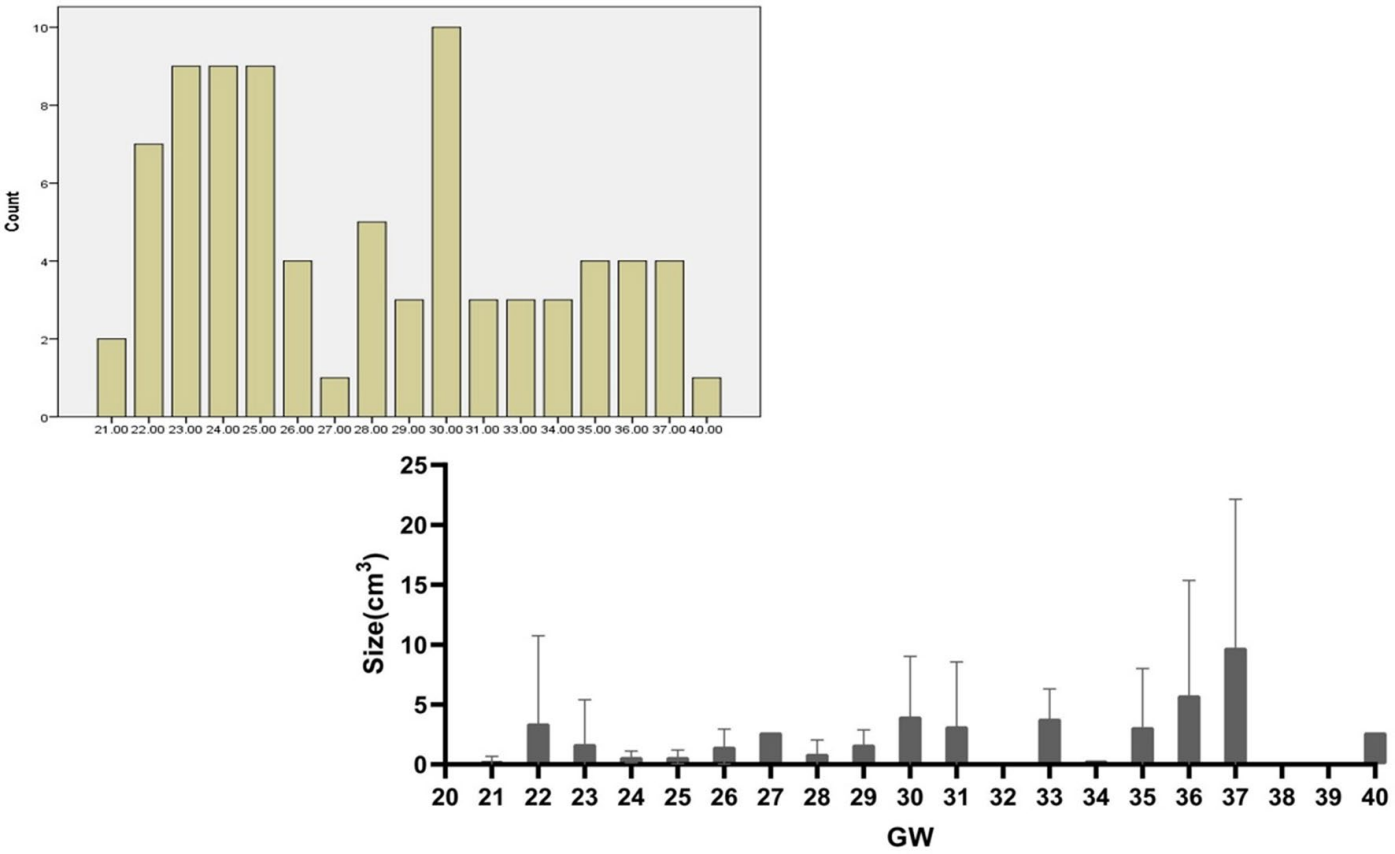
The flowchart of the recruited samples in this study.

The original Article has been corrected.

