# Supplementary material for: A computerized diagnostic model for automatically evaluating placenta accrete spectrum disorders based on the combined MR radiomics-clinical signatures
Source: Sci Rep. 2022 Jun 16;12:10130. doi: 10.1038/s41598-022-14454-w (PMC9203504; doi:10.1038/s41598-022-14454-w)
Supplement: Supplementary file 1 — Supplementary Information. [file 41598_2022_14454_MOESM1_ESM.docx]

**Table**

| **Supplementary Table 1. The detailed scan parameters across three MR machine.** | | | | | | | | | |
| --- | --- | --- | --- | --- | --- | --- | --- | --- | --- |
| **3T Philips** | | | | | | | | | |
| sequence | acquisition | Flip angle  （°） | Exciting number | TR/TE  (ms) | FOV(mm^2^) | resolution  (mm^2^) | Slice thickness/  gap  (mm) | slices | Scan time |
| TSE-SSH | SE-TSE | 90 | 1 | 10000/104 | 308×399 | 1.1×1.45 | 4.0/0 | 25 | 0 min 30s |
| BFFE | FFE-TFE | 90 | 1 | 2.8/1.41 | 383×387 | 1.5×1.49 | 4.0/-2.0 | 40 | 1 min 31s |
| T1WI | FFE-TSE | 90 | 1 | 10/2.3 | 375×305 | 1.6×2.15 | 7.0/0 | 30 | 1 min 01s |
| BSSFP | FFE | 90 | 1 | 3.3/1.65 | 350×350 | 1.8×2.3 | 5.0/0 | 1 | 0min 24 s |
| **1.5 T SIEMENS** |  |  |  |  |  |  |  |  |  |
| T2WI | HASTE | 170 | 1 | 1350/92 | 400 | 1.4× 1.1 ×4.0 | 4/0.8 | 15-20 | 20-25s |
|  | True-FISP | 60 | 1 | 3.87/1.68 | 400 | 1.7× 1.6 ×4.0 | 4/0.4 | 15-20 | 10-20s |
| **1.5 T GE** |  |  |  |  |  |  |  |  |  |
| T2WI | TSE | 50 | 2 | 3000/61 | 360 | - | 8/6 | 15-20 | 20S |


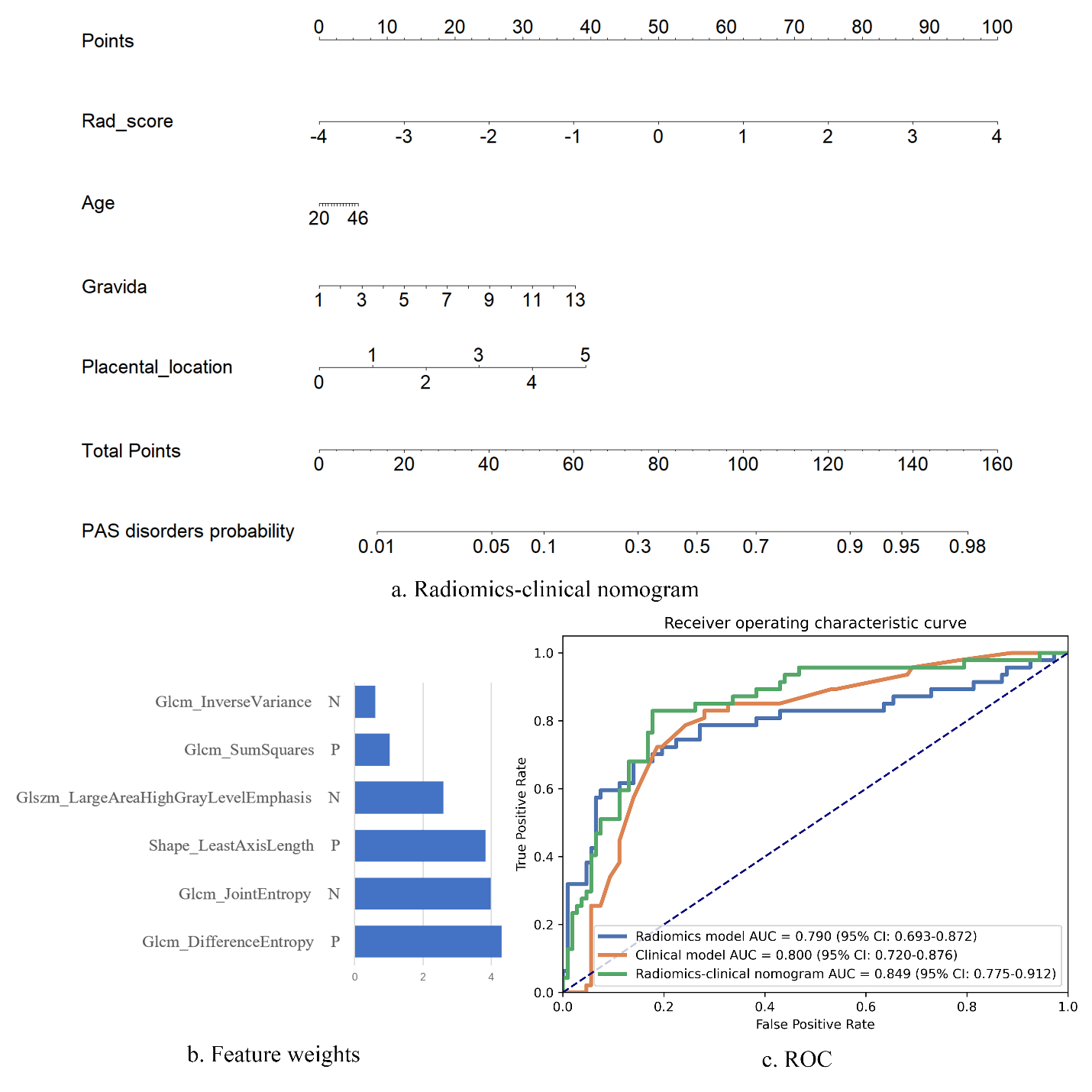


**Figure 1**. (a) The T2W radiomics-clinical nomogram. (b) The selected 6 radiomics features and their corresponding coefficients in the radiomics model. (c) The ROC curves of different models in testing cohort.
